# Characteristics of the Oxidative Status in Dairy Calves Fed at Different Milk Replacer Levels and Weaned at 14 Weeks of Age

**DOI:** 10.3390/antiox10020260

**Published:** 2021-02-08

**Authors:** Katharina Diana Seibt, Morteza Hosseini Ghaffari, Theresa Scheu, Christian Koch, Helga Sauerwein

**Affiliations:** 1 Institute of Animal Science, Physiology Unit, University of Bonn, Katzenburgweg 7, 53115 Bonn, Germany; katharina.seibt@uni-bonn.de (K.D.S.); morteza1@uni-bonn.de (M.H.G.); 2 Educational and Research Centre for Animal Husbandry, Hofgut Neumuehle, 67728 Münchweiler an der Alsenz, Germany; T.Scheu@neumuehle.bv-pfalz.de (T.S.); C.Koch@neumuehle.bv-pfalz.de (C.K.)

**Keywords:** dairy heifer, calf, oxidative status, development, adiponectin, leptin, haptoglobin

## Abstract

A paradigm shift in the way of rearing heifer calves from restricted feeding and early weaning towards greater feed allowances and later weaning ages is ongoing. We aimed at characterizing the oxidative status in Holstein heifer calves fed with milk replacer (MR) at either a restrictive (RES) or a high (HIGH) level for 14 weeks. We compared two groups: HIGH (10 L MR/d, n = 18) and RES (5.7 L/d, n = 19) from day five until week 14 of life. In blood samples collected at birth, and then fortnightly from week 8–16, and in week 20, the antioxidative capacity measured as ferric reducing ability of plasma (FRAP), oxidative damage of lipids measured as thiobarbituric acid reactive substances (TBARS) and oxidative damage of proteins measured as advanced oxidation products of proteins (AOPP), free radicals measured as reactive oxidative metabolites (dROM), and the glutathione peroxidase (GSH-Px) activity, as well as leptin, adiponectin and haptoglobin were assessed. The time course of these variables during the first 20 weeks of life showed characteristic patterns; group differences were limited to adiponectin, AOPP, and FRAP. RES calves had lower growth rates, showed signs of hunger, but did not differ from HIGH in their intake of solid starter feed and in health status. This work characterizes the changes in oxidative status of dairy calves with increasing age and confirms the benefits of a high feeding plane with regard to welfare and development.

## 1. Introduction

The development of dairy heifers in early life is considered as one of the most influential factors for the future health and profitability of dairy cows [1]. Growth and development are mainly affected by energy intake and the genetic potential of a calf [2]. Calves are born as functional monogastrics and their nutrient and energy intake is initially based on the intake of whole milk or milk replacer (MR) until transformation to mature ruminants [3,4,5].

If a calf is reared with its dam, the calf receives whole milk ad libitum, and up to 12 L/d are consumed divided into 8 to 12 meals/d [6,7]. The natural weaning process occurs gradually over several weeks at approximately 10 months of age [8,9]. In contrast, it is still common practice to feed dairy calves restrictively, i.e., 4–6 L/d, or 10% of body weight (BW) [10] and they are commonly weaned at 8 weeks of age (2 months) [2] to stimulate solid feed intake, which should advance rumen development [3] and thus reduce rearing costs by achieving greater weight gains from starter feed, which, in turn decreases age at first calving [2]. Hence the weaning process in conventional dairy farming is conflicting in several aspects with the natural weaning process.

However, there is growing evidence that increasing the daily allowance of MR for calves is superior to the restricted intakes in terms of growth, development [5,11] and also of welfare, by reducing hunger and avoiding abnormal behaviors [12]. Furthermore, an increasing daily MR allowance supports greater BW gain, improved feed efficiency, reduced incidence of disease and greater opportunity to express natural behaviors, which in combination suggest improved welfare [13]. An intensive preweaning feeding regimen, i.e., an increase of the daily allowance of MR to amounts that are close to calves’ ad libitum intake (10–14 L/day) [5,11] augments average daily gain (ADG) in calves which seems to promote milk yields in the first lactation [1].

Not only the plane of milk feeding is important, but also the weaning age. The calves’ energy intake via milk is crucial for development until they are mature ruminants. In conventional rearing, calves are weaned at 8 weeks (wk) of life, but the number of studies with weaning ages up to 10 wk of life increased in the past decades [2,14], but there are just a few studies on later weaning ages around 11 wk of life [15] and 14 wk [7] or 17 wk of life [16]. These studies consistently showed that delaying the weaning age is beneficial for development and reduces weaning stress by reducing the drop in energy intake and the behavioral signs of hunger that commonly occur during weaning [6,15]. The effects of different planes of nutrition in extended preweaning periods are thus less explored, and data about the oxidative status in calves until weaning and thereafter are limited [17]. Some studies focused on newborn calves [18,19] or tested the effect of supplementing vitamins or minerals on the calves’ antioxidant system around weaning [20]. In order to extend the existing knowledge, our objectives were to test for potentially positive effects of a high allowance for MR (HIGH, 10 L/d) as compared to restrictive MR allowance (RES, 5.7 L/d) for 14 wk MR feeding on growth and health, and to characterize the oxidative status until weaning and postweaning until wk 20 of life. We therefore assessed the effects of a high and restrictive milk replacer allowance on feed intake, feeding behavior, weight gain, health status and the circulating concentrations of the acute phase protein haptoglobin (Hp) of two adipokines, i.e., adiponectin and leptin, which are known to be associated with insulin responsiveness and body fat content, respectively. For characterizing systemic oxidative status, we addressed both the damage caused by oxidative stress on lipids (measuring thiobarbituric acid reactive substances, TBARS) and on proteins (assessing advanced oxidation products of proteins, AOPP). In addition, antioxidant activity (measured as ferric reducing ability of plasma, FRAP), the antioxidant selenoenzyme glutathione peroxidase (GSH-Px) as well as pro-oxidants (derivatives of reactive oxygen metabolites, dROM), were investigated.

We hypothesized that HIGH fed calves in comparison to RES would show higher rates of development without extreme drops after weaning, fewer signs of hunger and good health condition. We further assumed that feeding high planes of MR would reduce weaning stress and also alter the blood profiles of markers of oxidative status, as well as of Hp, leptin, and adiponectin.

## 2. Materials and Methods

The present study was conducted at the Educational and Research Centre for Animal Husbandry, Hofgut Neumuehle, Muenchweiler a. d. Alsenz, Germany, following the guidelines of the German Law for Animal Welfare by permission of the local authority in charge (G 17-20-071; Landesuntersuchungsamt Rheinland-Pfalz, Koblenz, Germany).

### 2.1. Animals, Diets, Feeding and Management

Thirty-seven German Holstein heifer calves were studied from birth until wk 20 of life, from February 2018 until March 2019. To ensure a controlled colostrum intake within the first three hours of life, the parturitions were monitored by a calving sensor system Moocall (Moocall LTD, Bluebell, Dublin, Ireland). Only healthy calves born without dystocia were included in this study. At least 3 L colostrum of the respective dam were administered via bottle to each calf within the first 3 h after birth. If a calf was not drinking the whole amount colostrum by itself, it was drenched. In the case of insufficient colostrum quantities available from the dam (n = 4), high-quality colostrum stored at −20 °C was added to reach the amount of 3 L. The quality was assessed with a Brix refractometer (Digital hand-held PAL-S refractometer, ATAGO, Tokyo, Japan) and specific gravity (SG) by a spindle (Kruuse Colostrum Densimeter, Kruuse UK Ltd., Langeskov, Denmark). The total protein content in calves´ serum within 48 h of life was also assessed by optical refractometer measurements (Euromex Microscopen bv, Arnhem, The Netherlands). Each calf received 10 mL of an iron suspension per oral administration (Sinta fer-o-bac, 115 mg Fe ^3+^/mL, Sinta GmbH, Schwarzenborn, Germany), and navels were disinfected with a 10% povidone-iodine solution (Vet-sept solution, aniMedica GmBH, Senden-Bösensell, Germany) or Engemycin^®^ spray (Intervet Productions Srl., Aprilia, LT, Noale, Italy) to protect against bacterial infections.

From birth until day 10 of life, the calves were kept in straw-bedded single hutches and were fed manually twice per day with teat buckets at the regular feeding times of 12 h intervals. Until the 9th meal (day five of life) all calves received 5 L twice per day of their dam’s acidified transition milk (2 mL/L of Schaumacid acidifier, H. Wilhelm Schaumann GmbH, Pinneberg, Germany) in the morning and evening, respectively. Transition milk was also supplemented with a mix of trace elements and vitamins (1 mL/L Milkivit Quick- Mix, Trouw Nutrition Deutschland GmbH, Burgheim, Germany). Thereafter the calves were assigned to two different feeding groups considering their birth weight, sire, and parity of the dam to create equal groups. Half siblings were also considered and were equally distributed on the feeding groups. From the 10th meal (day five of life) onwards calves received milk replacer (MR; 14% solids; Milkivit Titan, Trouw Nutrition Deutschland GmbH) either at 10 L/d (HIGH; 1.4 kg MR powder/d; n = 18) or at 5.7 L/d (RES; 0.8 kg MR powder/d; n = 19). During this time, from birth until the 20th meal (day 10 of life), the calves were kept in straw-bedded single hutches and the intake of transition milk and MR was documented by weighing the amount in buckets before feeding and the residues in the bucket before offering the next meal. While the calves were in single hutches, they were provided with fresh water ad libitum in buckets and small hayracks every day. Afterwards, at day 10 of life, the calves were moved to an open straw-bedded stable where they were kept in a group. Newly incoming calves were assigned to a “baby group”, in a separated compartment within the stable for the first five days. This “baby group” was created for enabling the calves to learn to suckle and drink on the automated feeding system without having to compete with older calves.

In the stable, from day 10 (wk 2) to day 98 (wk 14) of life, an automated feeding system (Vario Kombi, Förster-Technik GmbH, Engen, Germany) for calf starter concentrate and MR ensured milk feeding according to respective feeding regimen. The intake of both MR and pelleted calf starter concentrate (Blattin Kälberstart Gold, Höveler Spezialfutterwerke GmbH & Co. KG, Dormagen, Germany), as well as the number of rewarded and unrewarded visits and the drinking speed were recorded individually by the feeding system and its software (Förster-Technik GmbH). Water, hay and calf starter were offered ad libitum. The daily water intake was not recorded, neither in single hutches nor in group pens. The ingredients and the chemical composition of the MR and starter (concentrate) are given in Table 1. At day 15 of life, the calves were moved from the “baby group” to group pens that were also equipped with an automated feeding system (Förster-Technik GmbH) until weaning starting at day 84 (wk 12 of life). In week five5 of life, all calves were dehorned under sedation and with local anaesthesia. The weaning process comprised a gradually reduction of daily MR allowance (by 0.3 L/d in RES and by 0.6 L/d in HIGH) over 2 weeks, i.e., until day 98 (week 14) of life, i.e., the last two days of MR feeding the maximum amount was 2.6 L/d in HIGH and 2.2 L/d in RES and on the last day for both feeding groups 2 L/d. From day 99 onwards the MR supply was stopped (0 L/d) and calves were moved to a new group pen in another stable where they had free access to water and a total mixed ration (TMR) for heifers (Table 1).

### 2.2. Records of Health Status and Body Weight

Birth weight was measured after colostrum intake whereby the amount of ingested colostrum was subtracted. The health status was checked weekly by veterinarians over the entire 20 weeks of the trial. On the day of the weekly health check, body weight (BW) was recorded until week 20 of life using a mobile electronic scale (KWM GmbH Waagen- und Metallbau, Thiersheim, Germany). The variables assessed during the health check and the score assigned are summarized in the Supplementary Table S1. The health check data of all time points and each calf were categorized in one summarizing health score as follows: 1 = checks without any health disturbances and thus entirely healthy, 2 = checks with one health disturbance, but not diseased, 3 = checks indicating disease and/or >40 °C rectal temperature, and 4 = checks with rectal temperature >39.5 °C without any other symptoms indicating disease or disorders. Health disturbances or appearing cases of diseases were further categorized as: 1 = rectal temperature > 39.5 °C, 2 = digestion, 3 = respiration, 4 = eye-related, and 5 = navel infection. In the case of health disturbances, such as diarrhea and infection of the navel, the calves were treated by a veterinarian and the incident was documented.

### 2.3. Calculation of Average Daily Gain (ADG) and Energy Intake

The ADG was calculated by dividing the weekly weight gain by the number of days between each weighing. The daily intake of metabolizable energy (ME) via MR and concentrate was calculated by multiplying the individual daily intake of each calf by the ME content (MJ/kg of DM) of MR (18.6), and starter concentrate (11.2). The ME content of MR was calculated using the equation: ME, MJ/kg of DM = (24.2 × CP + 36.6 × fat + 17.0 × total sugar)/100 × 0.97 gross energy (GE) × 0.96 digestible energy (DE), as detailed by Frieten et al. (2017) [11] and the National Research Council [21].

### 2.4. Blood Sampling and Analyses

Blood samples were taken by jugular vein puncture from 36 calves seven time points, i.e., 36–48 h after birth, from week 8 until week 16 of life in two-week intervals (week 8, 10, 12, 14, 16), and in week 20 of life. Out of a total of 37 calves, blood samples were taken from only 36, because one calf failed to complete the sampling schedule and was therefore excluded. This calf was neither diseased nor dead. Blood samples were also collected from the respective dams by coccygeal vein puncture 36–48 h after calving. Blood was collected in 10 mL serum and EDTA-plasma tubes (Sarstedt, Nümbrecht, Germany). The EDTA-plasma samples were centrifuged at 4 °C for 20 min at 3000× *g*, whereas serum tubes were incubated for 45 min at room temperature (RT) for clotting before centrifugation for 10 min at 3000× *g* at room temperature. Afterwards, serum and plasma were aliquoted and stored at −20 °C until analysis. The total protein content in the first serum samples (36–48 h after birth/post natum (p.n.)) was determined by an optical refractometer (Euromex MicroscopenB.V., Arnhem, Netherlands). Haptoglobin (Hp), leptin and adiponectin in serum were measured using in-house developed ELISAs [22,23,24]. The mean intra and interassay coefficients of variation (CV) were 9.9% and 12.1% for Hp, 6.7% and 11.3% for leptin, and 9.6% and 11.2% for adiponectin, respectively.

### 2.5. Measurements of the Oxidative Status in Plasma

Reactive oxidative species were measured using the dROM test (detection of reactive oxygen metabolites) according to Alberti et al. (2000) [25] with modifications [26] using N, N-diethyl-para-phenylendiamine as chromogenic substrate. The dROM values are expressed as H_2_O_2_ equivalents. The mean intra-assay CV was 2.11% and the interassay CV was 10.6%. The antioxidative capacity was assessed as FRAP (ferric reducing ability of plasma) according to Benzie and Strain (1996) [27] with a mean intra-assay CV of 2.94% and an interassay CV of 5.20%. The values are given as μmol Fe2^+^/L. The concentrations of AOPP (advanced oxidation products of proteins) were measured according to Witko-Sarsat et al. (1996) [28], with a mean intra-assay CV of 1.24% and the interassay CV of 3.16%. The AOPP values are expressed per volume unit (μmol/L) as well as per g of total protein (μmol/g). For this purpose, the total protein concentration in plasma was assessed by the Bradford method [29] using BSA as standard. The mean intra-assay CV was 1.83% and the mean interassay CV was 5.56%. In addition, the TBARS (thiobarbituric acid reactive substances) test [30] was used for measuring oxidized lipids in plasma with a mean intra-assay CV of 6.18% and a mean interassay CV of 9.08%. The concentrations are given as nM malonyldialdehyde (MDA) which was used as standard. The activity of the selenium-dependent glutathione peroxidase (GSH-Px) in plasma was determined spectrophotometrically according to original method proposed by Paglia and Valentin (1967) [31]. One unit (U/mL) equates to the amount of enzyme necessary to catalyze the oxidation of 1 μmol NADPH per min at 25 °C, pH 7.0. The mean intra-assay CV was 4.04% and the mean interassay CV was 4.40%.

### 2.6. Statistical Analyses

Data were statistically analyzed using SPSS (version 26; SPSS Inc., Chicago, IL, USA). The normal distribution was checked by the Kolmogorov-Smirnov-test and the homogeneity of variance with the Levene’s test. If the data were not normally distributed, nonparametric tests were performed and data were log-transformed for further statistical analysis. For the identification of outliers in metabolic hormones, Hp, and oxidative parameters, box plots of each raw data set were checked, and Z-standardization was performed. Outliers in total protein, AOPP, TBARS, dROM, FRAP, GSH-Px, Hp, and leptin were identified as values with SD > 2, in Adiponectin with SD > 3 (Z-standardization) and were excluded. Furthermore, data were analyzed by a linear mixed model with Bonferroni post hoc tests and with repeated measurements, considering the individual calf as a random effect and feeding group (group), and week of life (time) and group by time interaction as fixed effects. Differences between the groups within each time point were tested with Student´s t-test or Mann-Whitney-test if data were not normally distributed and homogeneity of variance was not given. Health check score categories in the two feeding groups were compared by the Chi-square test. Correlations (ρ) were calculated by Spearman analysis. Results were declared as significant when *p* < 0.05, and 0.05 ≤ *p* < 0.10 was considered as a trend. In all graphs, non-transformed data are shown as means ± SEM.

## 3. Results

### 3.1. Birth Weight and Colostrum Intake

Within the first 2 h after birth, each calf received at least 3 L of colostrum. In total, 28 calves drank the whole amount of offered colostrum by themselves, five calves had to be drenched and four calves have been additionally drenched the rest of the colostrum to reach the whole amount of 3 L. The following data are reported as means ± SD. Birth weight in group HIGH (10 L/d, n = 18; 39.7 kg ± 6.0) was not different (*p* = 0.472) as compared to group RES (5.7 L/day, n = 19; 41.0 kg ± 4.1). The first colostrum intake was similar in both groups (HIGH 3.3 kg ± 0.8; RES 3.3 kg ± 0.6; *p* = 0.327). Colostrum quality was not different between the groups: The mean Brix values in HIGH were 23.5% ± 3.9 and 25.3% ± 3.9 in RES, respectively (*p* = 0.209). Based on the measured Brix values of >22% and at least 3 L colostrum intake in all calves, a supply of at least 150 g IgG with the first meal was expected [32]. The second meal of dams’ milk (5 L) was fed no later than 12 h after birth. The specific gravity (SG) as assessed by spindle was >1045 in both groups, and thus colostrum was classified as good quality according to the reference zone on the spindle provided by the manufacturer (Kruuse UK Ltd., Langeskov, Denmark). However, the spindle values in the HIGH group were slightly lower than in the RES group (HIGH 1047 SG ± 5.7 vs. RES 1052 SG ± 4.7; *p* = 0.002). The protein concentration (g protein/100 mL) in serum, 36 - 48 h after birth, measured by optical refractometer, was similar between both groups and thus confirmed an adequate intake of colostrum of good quality (HIGH 5.4 ± 0.5; RES 5.6 ± 0.7; *p* = 0.377).

### 3.2. Intake of Milk, Starter, and Metabolizable Energy (ME)

**Figure 1:**
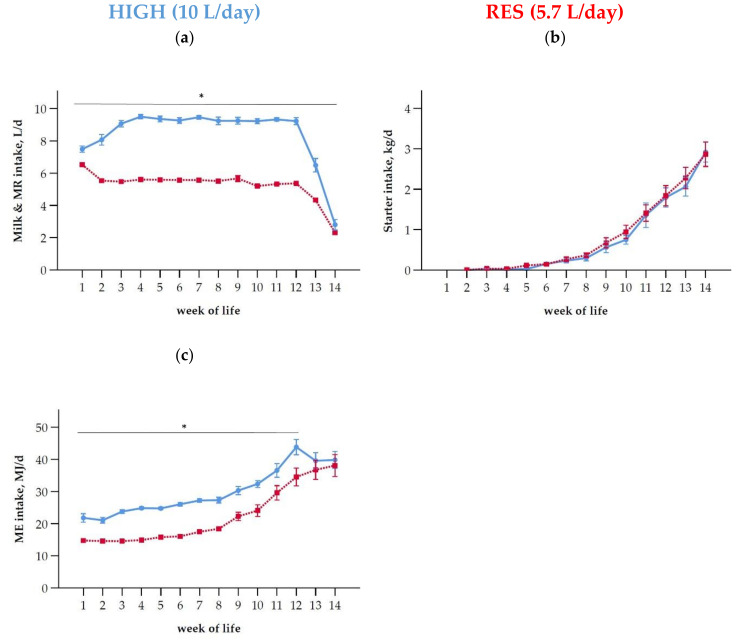
Preweaning intakes (means ± SEM) of (**a**) transition milk (whole milk of the dam, first 5 d of life) or/and milk replacer (MR), (**b**) starter, and (**c**) of metabolizable energy (ME) of calves with preweaning high allowance to MR (HIGH; blue line) or restrictive allowance (RES; red line). The MR intake was gradually reduced in week 13 and 14 to 2 L/d in both groups. Asterisks indicate differences (*p* < 0.05) between groups within individual time points (week of life).

The daily milk and MR intake differed between the two groups throughout the differential liquid feeding period from day five until the end of weaning at 14 weeks of age (Figure 1a, Table 2). The data reported in this text section are means ± SD. During the first five days of life, milk intake was 7.1 L/d ± 2.4 in the HIGH group and 6.9 L/d ± 2.3 in the RES group, without differences between the groups (*p* = 0.536). From week 2 (beginning of differential feeding) until the end of week 12 (onset of weaning), the RES calves consumed 5.5 ± 0.4 L MR/d, whereas the HIGH calves drank 9.2 L/d ± 0.9 L MR/d (Figure 1a). The mean starter intake was not different between both groups over the entire milk-drinking period until week 14 (Figure 1b, Table 2) and was negligible in both feeding groups in the first 6 weeks of life. Thereafter it increased to 2.9 kg/d ± 1.2 kg/d in wk 14 at the end of weaning. Figure 1c shows the ME intake of both feeding groups. HIGH had greater daily ME intakes than RES over 12 weeks of life until the beginning of weaning. This difference leveled off during the weaning process (weeks 13 and 14 of life, Figure 1c).

### 3.3. Rewarded and Unrewarded Visits

**Figure 2:**
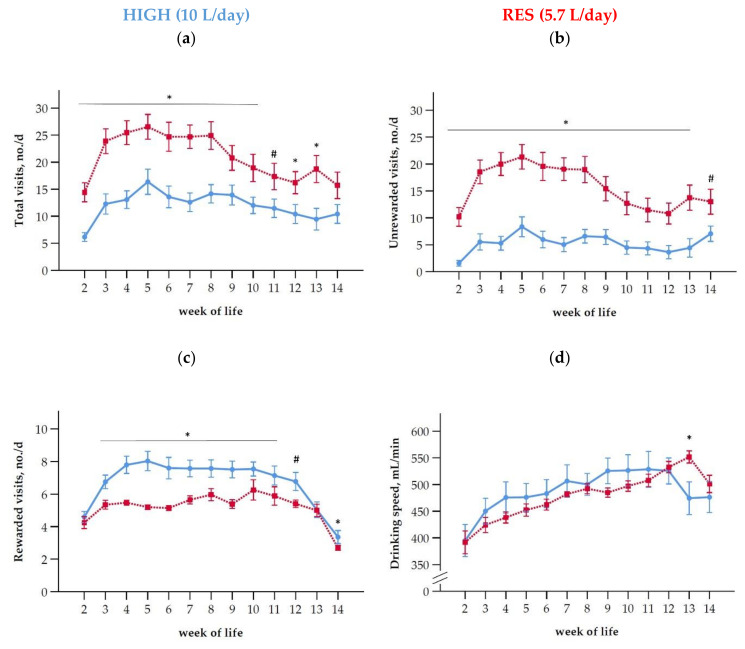
Preweaning number (means ± SEM) of (**a**) total visits at the automatic milk feeding station per day (no./d), (**b**) number of unrewarded, (**c**) rewarded visits, and (**d**) drinking speed in calves fed at a high level of MR (HIGH; blue line) or had a restricted MR allowance (RES; red line). For weaning, MR intake was gradually reduced in weeks 13 and 14 to 2 L/d in both groups. Asterisks indicate differences between groups within week of life (*p* < 0.05) and hashtags indicate trends (*p* < 0.1).

Over the entire liquid feeding period, the RES group visited the automatic milk feeder more often per day than the HIGH group (Figure 2a, Table 2). In week 5, the number of visits/day (means ± SD) peaked in both groups, whereby RES had 1.6-fold more visits than HIGH (26.5 ± 10.1 vs. 16.4 ± 9.6; *p* = 0.004). The RES calves also had more unrewarded visits over the entire time until week 14 of life (Figure 2b, Table 2). From week 3 until week 11, HIGH calves had more rewarded visits than RES (Figure 2c). During the linear weaning, in week 13 (*p* = 0.76), but not in week 14 (*p* = 0.023), the number of rewarded visits per day was not different between HIGH and RES anymore (Figure 2c), while unrewarded visits were still higher in RES (Figure 2b). In both groups, the drinking speed was increasing with age until week 12 and was not different between the groups (Figure 2d, Table 2). During weaning the situation changed and RES calves showed a higher drinking speed (mL/min, means ± SD) in week 13 than HIGH (551.7 ± 49.5 vs. 474.4 ± 122.8; *p* = 0.01), but not in week 14 (*p* = 0.76, Figure 2d).

### 3.4. BW and ADG

**Figure 3:**
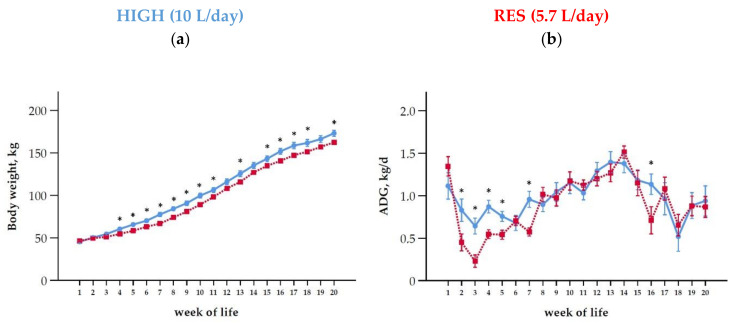
Pre and postweaning changes (means ± SEM) of (**a**) body weight and (**b**) average daily gain (ADG) of calves fed at a high level of MR (HIGH; blue line) or at a restricted level (RES; red line). Weaning was done by gradually reduced the MR allowance from week 13 to 14 to 2 L/d in both groups. Postweaning, calves were moved to a new group pen in another stable where they had free access to a TMR (Table 1). During the whole trial the animals had free access to water and hey. Asterisks indicate differences between groups within week of life (*p* < 0.05).

On average, HIGH calves were heavier than RES over the entire observation period until 20 weeks (Figure 3a, Table 2). In none of the groups, weaning was associated with BW loss. Overall, both groups gained steadily in weight. The group differences in BW were significant from week 4 to 11, in week 13 during weaning, and in weeks 16–18 and 20 after weaning (Figure 3a).

The ADG fluctuated over the trial period and was affected by group, time, and the interaction thereof (Figure 3b, Table 2). On average, the ADG decreased until week 3 and increased until the end of weaning (week 14). Calves of HIGH gained more than RES from weeks 2–5 and in week 7 of life (Figure 3b). After weaning, calves were transferred to another stable with older calves and received a TMR (Table 1). After changing stables in week 15, a decline in ADG was observed in both feeding groups, which was more pronounced in the RES calves (1.5-fold lower) than in the HIGH ones in week 16, i.e., 2 weeks after weaning (Figure 3b).

### 3.5. Health Records

Throughout the 20-week observation period, 23 calves (62.2%) remained entirely healthy without any clinical signs. In general, the incidence of health checks indicating a diseased calf (and/or >40 °C rectal temperature) within the entire 20 weeks of the trial was low: In 2.97% of all 740 health checks (22 findings in total), indications for a disease were observed: 14 animals (37.8%) were diseased once or more often, whereby the diseased animals were equally distributed between the two feeding groups, i.e., seven calves each. In week 6 of life after dehorning in week 5, the incidence of cases of fever or elevated body temperature (>39.5 °C) peaked. In the summarizing health score categories (Supplementary Table S1) of both groups, there was a trend (*p* = 0.073) for differences between groups, i.e., in HIGH 86.7% of all checks were in health score category 1 (completely healthy) and in RES calves 92.4% of all checks. In the further categorization of the occurring health disturbances and diseases no difference appeared between feeding groups.

### 3.6. Haptoglobin (Hp)

**Figure 4:**
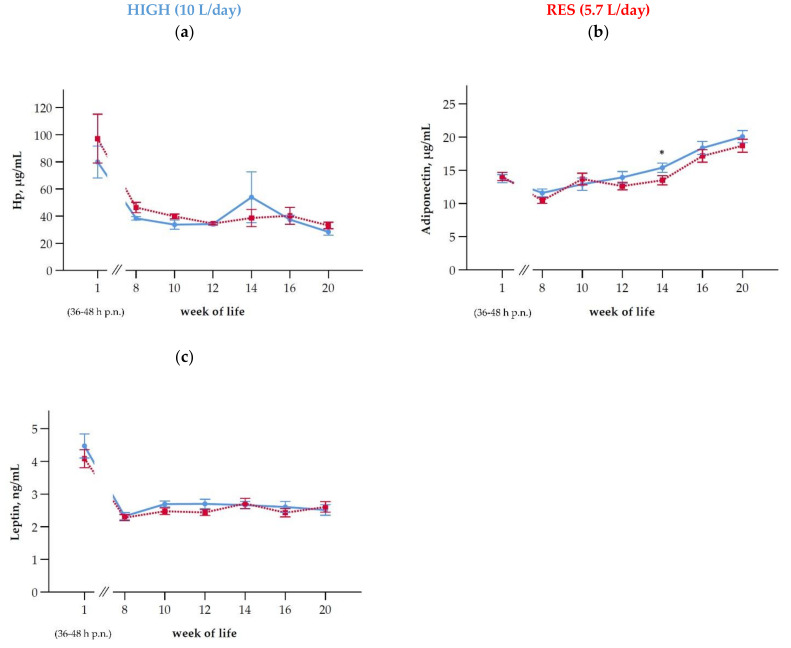
Time-dependent changes (means ± SEM) of (**a**) haptoglobin (Hp), (**b**) adiponectin, and (**c**) leptin in calves fed at a high level of MR (HIGH; blue line) or a restrictive level (RES; red line). The MR intake was gradually reduced in week 13 and 14 to 2 L/d in both groups. From week 15 until week 20 of life, the calves were moved to a new group pen in another stable receiving a TMR (Table 1). Asterisks indicate differences between groups within weeks of life (*p* < 0.05) and hashtags indicate trends (*p* < 0.1). After birth/*post natum* is abbreviated as p.n.

When considering all Hp values, there were neither time nor group effects, and also the interaction was nonsignificant. In total, 18 extreme Hp outliers (SD > 2; Z-standardization within each time point) were identified. By excluding these 18 outliers, there was a time effect (*p* < 0.001), but the feeding group had no effect (Figure 4a, Table 3). The extreme outliers (SD > 2 and Hp > 1000 µL/mL) were mainly observed in week 16 (n = 3, RES) and in week 20 of life (n = 4; equally in RES and HIGH). Additionally, in week 20, two outliers (one each in RES and HIGH) with 400 µL/mL < Hp < 1000 µL/mL were excluded. The Hp concentrations recorded in week 16 of these three calves (RES) were >4000 µg/mL. These outliers, which were observed as elevated Hp values, could not consistently be attributed to clinical findings: Only one calf of the three calves with elevated Hp in week 16 had a fever and one calf of the six calves with elevated Hp in week 20 had an elevated rectal temperature (39.6 °C), whereas the remaining ones showed no signs of disease. Similarly, for the outliers identified in week 1 to week 14 (n = 9), concurrent clinical findings were limited to one calf, which showed a slight navel infection without fever or other health disturbances. It should be noted that health checks were done independently of the blood sampling. After birth (36–48 h), Hp concentration in the serum of calves were lower than in the serum of their respective dams after calving (*p* < 0.001), irrespective of the feeding groups (Table 3).

### 3.7. Metabolic Hormones

The concentrations of adiponectin in serum were affected by both group and time, without the respective interaction. Three outliers in adiponectin (SD > 3, Z-standardization) were observed in week 8, 10, and 12. Higher values were observed 36–48 h after birth (wk 1 of life) in both groups (13.9 ± 2.61 µg/mL; mean ± SD) than in week 8 (11.0 ± 2.18 µg/mL, *p* < 0.001), whereas there was no difference between week 1 and week 10, 12, and 14, respectively (Figure 4b). From week 14 onwards, the adiponectin concentrations were steadily increasing until week 20 (Figure 4b). In week 14 higher adiponectin concentrations in HIGH than RES appeared (*p* = 0.048; Figure 4b). In addition, the correlations between adiponectin and Hp and the parameters of the oxidative status were determined: Between adiponectin and Hp, there were no association detectable (ρ = −0.044, *p* = 0.509). Considering the indicators for oxidative status tested herein, there were weak correlations between adiponectin and AOPP (µmol/g of protein; ρ = −0.135, *p* = 0.037) and dROM (ρ = 0.376, *p* < 0.001) detectable, but not between adiponectin, FRAP and TBARS, respectively (ρ = −0.077, *p* = 0.237; ρ = −0.094, *p* = 0.152).

The leptin concentrations decreased from week 1 until week 8 of life (*p* = 0.001) and remained rather stable from week 8 to 20 of life without any group differences (Figure 4c). One calf in the HIGH group had considerably greater leptin concentrations than the mean from all calves in five out of seven time points, which were further recognized as outliers (SD > 2) and therefore excluded from further statistical analysis. When comparing the hormone concentrations between the calves and their dams, about 5-fold higher leptin concentrations were observed in cows than in their calves (*p* > 0.001; Table 3). For serum adiponectin the situation was vice versa: Calves had nearly 3-fold higher adiponectin concentrations than their respective dams (*p* < 0.001; Table 3). Neither the Hp, nor the leptin and adiponectin concentrations of the dams were correlated with those of their calves (36–48 h after calving; Hp ρ = 0.170, *p* = 0.361; Leptin ρ = −0.206, *p* = 0.249; adiponectin ρ = 0.216, *p* = 0.253).

### 3.8. Oxidative Status

**Figure 5:**
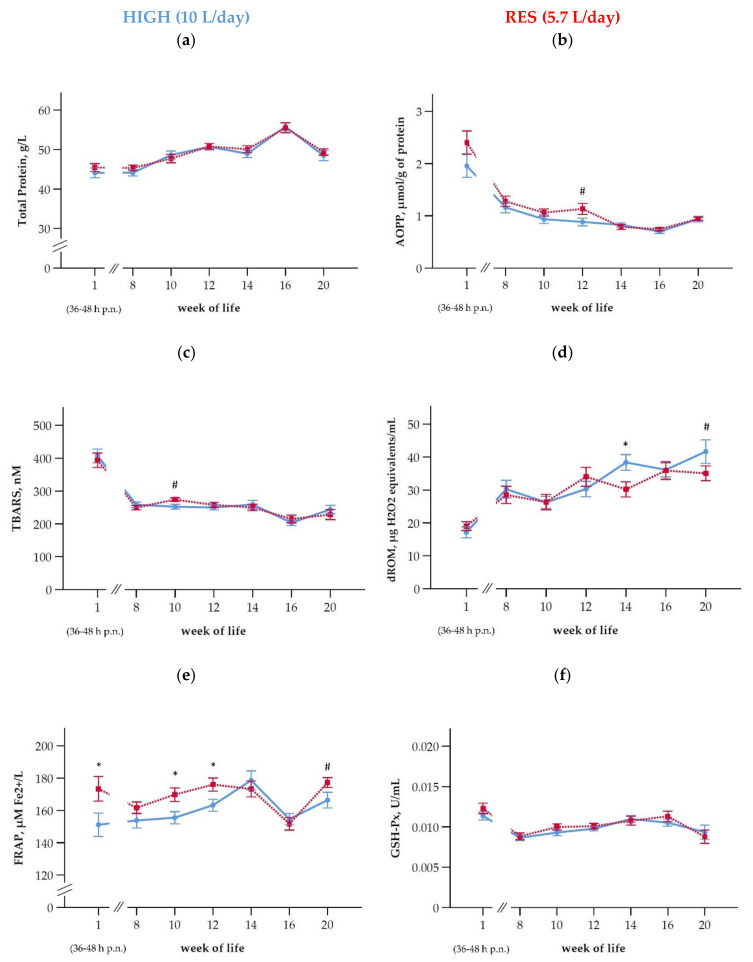
Changes in the systemic oxidative status during the first 20 weeks of life (means ± SEM): (**a**) Total protein content in plasma, (**b**) advanced oxidation products of proteins (AOPP), (**c**) thiobarbituric acid reactive substances (TBARS), (**d**) reactive oxidative metabolites (dROM), (**e**) ferric reducing ability of plasma (FRAP), and (**f**) glutathione peroxidase activity (GSH-Px) in calves fed at a high level of MR (HIGH; blue line) or at a restricted level (RES; red line). The MR intake was gradually reduced in wk 13 and 14 to 2 L/d in both groups. From week 15 until week 20 of life, the calves were moved to a new group pen in another stable receiving a TMR (Table 1). Asterisks indicate differences between groups within wk of life (*p* < 0.05) and hashtags indicate trends (*p* < 0.1). After birth/*post natum* is abbreviated as p.n.

The total protein concentration in plasma, assessed as reference for the AOPP values, but also as a general indicator of health, was not different between groups, but increased with time until week 16; in week 20 decreasing protein concentrations were observed (Figure 5a,b and Table 3). Regardless of being considered per volume or per g total protein, the AOPP values were mainly influenced by time, but also by feeding group (Figure 5b, Table 3). In the first sample collected (36–48 h after birth), the highest AOPP values were recorded with a steady decline towards the end of the observation period (Figure 5b). The AOPP concentrations in calves were higher than those of their dams in the postpartum samples (Table 3). The TBARS values decreased with time but were not different between feeding groups (Figure 5c).

The dROM values in plasma increased with time but were not affected by the feeding regimen (Figure 5d, Table 3). Differences in dROM between both groups were limited to week 14 (*p* = 0.015) at the end of weaning and a trend for differences in week 20 of life (*p* = 0.05), respectively (Figure 5d). For FRAP, both group and time were significant; the RES calves had higher values than the HIGH calves in the preweaning period (Figure 5e, Table 3). From the end of the weaning procedure in week 14 until the end of the trial in week 20, the group differences were balanced out (Figure 5e).

In comparison to their respective dams after calving, the FRAP values in calves amounted to about 70% of those observed in their dams (Figure 5e, Table 3). The differences between the feeding groups of calves after birth when feed intakes were not yet different were not related to the dams’ FRAP status, as indicated by the equal values in the dams of both groups (Table 3) and the lack of correlations between calves and dams (FRAP ρ = −0.270, *p* = 0.135; AOPP ρ = 0.267, *p* = 0.133).

The activity of the selenium-dependent GSH-Px, which catalyzes the reduction of hydrogen peroxide (H_2_O_2_) with a simultaneous oxidation of glutathione, was not influenced by feeding group (Figure 5f, Table 3).

## 4. Discussion

### 4.1. Feed and ME Intake

Expectedly, MR intake was greater in HIGH than in RES calves during the entire time of the differential feeding preweaning. The average daily MR intake of 9.18 L in HIGH was less than the daily allowance of 10 L, in particular, during the first weeks of life, when the HIGH calves were still increasing their intake until they reached a plateau of about 9 −10 L/d around week four of life. These findings were similar to other reports [5,10,33]. Previous studies have demonstrated that MR-intake during weaning is less for late-weaned (12 or 16 weeks) calves compared to early-weaned (6 weeks) calves, confirming that with increasing age the demand for milk is less due to a substituting intake of energy via calf starter and hay [6,16]. De Passillé et al. (2011) [6] showed that later-weaned calves on a high milk feeding regimen ate more starter and hay and had higher energy intakes and weight gains, and made fewer visits to the automated milk feeder than early-weaned calves fed on a high milk feeding level.

The lower number of visits to the automated feeding station in late weaned calves reflects the reduced need for milk with increasing age and less hunger. In our trial, a decrease in the number of visits at the automated feeder from week eight onwards was registered in both groups, in particular in the HIGH group. The greater number of unrewarded visits at the automatic feeding station in the RES group in our trial supports the notion that this level of restriction is not satisfying hunger, which is in line with previous findings [5,14]. Thus, the conventional restricted feeding violates one of the principal features of animal welfare, i.e., the freedom of hunger as one of the five freedoms formulated by Webster (1997) [12]. Increased numbers of unrewarded visits in restrictively fed calves were previously reported [5,14]. At preweaning, both groups in our trial showed a steady increase in drinking speed with age. Surprisingly, the situation changed during weaning, when RES calves showed still an increase whereas the HIGH calves reduced their drinking speed. This result is in contrast to previous findings of Gerbert et al. (2018) [14] showing that ad libitum fed calves had lower drinking speeds than restrictively fed calves. De Passillé et al. (2011) [6] showed that delaying the weaning age can reduce a decline in energy intake and behavioral signs of hunger. Furthermore, our trial showed, that even if the weaning age is delayed to 14 weeks of age, the desire for milk remains.

It is well known that a close relationship between milk feeding and solid feed consumption of calves exists [13]. Therefore, greater starter intakes in RES for compensating the lesser energy intake via MR and satisfying the feeling of hunger might be expected, as reported also for calves weaned between 8 and 10 weeks of life [5,10,11,33,34]. However, both feeding groups, HIGH and RES, had equal starter intakes during the preweaning period. Accordingly, the ME intake was higher in HIGH calves than in RES until the beginning of weaning, when ME intake via MR was reduced, confirming results reported by Rosadiuk et al. (2020) [33]. Our findings are thus in support of the notion that a high plane of MR feeding does not counteract starter feed intake.

The consumption of starter is not only promoted by lesser milk or MR intake. Another main influencing factor is the developmental stage of the rumen and its ability to digest solid feed for an adequate energy supply. Therefore, weaning age also influences the consumption of starter and gradual weaning stimulates the starter intake during the preweaning period [13]. In our trial, the starter intake of calves increased mainly after week nine of life and this is the age in which most of the previous studies started or even finalized weaning. The higher number of unrewarded visits in the RES group even after week 9 of life showed that these calves were still in a developmental stage in which milk is an important and digestible source of energy and therefore better suited to satisfy hunger than starter concentrate and delivers energy as the basis for growth and development.

Previously, a high and early intake of starter was assumed to be associated with an enhanced rumen development, an earlier possible weaning age and lesser costs for milk replacer [2,5]. Korst et al. (2017) [5] calculated higher costs for ad libitum milk feeding than for restrictive feeding, but they concluded ad libitum feeding enhances animal welfare and later economic profit from milk. Therefore, the savings achieved by reducing MR costs should be balanced against the potential benefit of improved growth and welfare. Besides, this ratio is not constant and depends on the prices for rearing including MR and income from milk.

### 4.2. BW Gain and ADG

The HIGH feeding resulted in a greater pre and postweaning BW than in RES. The ADG declined in both groups within the first 3 weeks of life, whereas RES calves showed a stronger decline in ADG than HIGH calves. The restrictive feeding regimen thwarted the ADG and caused a slower BW gain in comparison to HIGH calves preweaning. Postweaning, ADG in both groups decreased but HIGH fed calves showed even then still a higher BW. These findings are in line with previous studies [5,10,11], confirming improved preweaning growth with enhanced milk or MR intake. Additionally, findings from Rosadiuk et al. (2020) [33] showed that the preweaning ADG and BW were greater for heifers offered 10 L milk/d, but, in contrast to our results, these effects were not maintained after weaning. Geiger et al. (2016) [35] showed that an enhanced preweaning MR feeding at 1.13 kg MR/d increased BW beyond weaning in week eight as compared to restrictively fed calves (0.45 kg MR/d). This corresponds to our results. Jasper and Weary (2002) [10] described that during and immediately after weaning the growth rate and BW gain slowed down independent of the MR feeding regimen.

### 4.3. Health Status and Hp

Haptoglobin (Hp) is a major positive acute-phase protein in cattle [36]. In newborn calves, the concentrations decrease during the first days of life when colostrum is provided [37], but there is no indication that colostral Hp is directly transferred into the calf’s circulation [37,38]. Both morbidity and mortality during the first 4 months of life were demonstrated to increase in calves with elevated Hp in the first week of life, but the predictive and diagnostic value of Hp measurements is limited [39]. In our study, we found little concordance between increased Hp concentrations and health disturbances.

It should be noted that the health checks were not necessarily performed at the same day of blood sampling. In our trial, we observed no differences in the number of diseased animals, the incidence of health disturbances, and the Hp concentrations between feeding groups. There were also no differences in the Hp values of the cows related to the group allocation of their calves. Nevertheless, the variation in Hp concentration in all calves was higher after birth than in the following weeks. The time course of the Hp concentration in the calves of both feeding groups of our trial, i.e., a decrease after the first wk of life to a constant level until the end of the trial, is in line with the results from Hulbert et al. (2011) [40].

Weaning may result in increased Hp concentrations as reported by Belli et al. (2018) [41] for calves fed ad libitum and weaned at 7 weeks of age. Hulbert et al. (2011) [40] showed that early-weaned calves (45 d) compared with later weaned calves (66 d) had increased plasma concentrations of cortisol and Hp, suggesting that weaning is less stressful when calves are older. In line with this, we did not see a distinct peak of Hp around weaning at 14 weeks of age in our trial. However, the sampling frequency might have been too low for detecting short-time changes.

### 4.4. Metabolic Hormones

Adiponectin and leptin are mainly secreted from adipose tissue and are thus termed adipokines [42,43]. Both hormones are involved in several physiological processes. Adiponectin is considered as an insulin sensitizer and is also involved in the regulation of lipid and glucose metabolism, as well as in inflammation [44]. In neonatal calves, the concentrations of both adiponectin and leptin were demonstrated to increase with colostrum intake within the first day of life [5,34,45,46]. In the present study, the first blood sample collected from the calves was collected after colostrum intake (36–48 h after birth), and the concentrations of both adiponectin and leptin were in the same range as reported previously [34]. There was no correlation between the calves’ leptin nor adiponectin serum concentrations with those of their dams.

Studies in humans have demonstrated inverse relations between circulating adiponectin and BW or body fat, in particular, visceral fat [47]. Decreasing concentrations of adiponectin were also reported in overfed dairy cows [48]. In the present study, the feeding level of MR affected the serum concentrations of adiponectin, which were greater in the HIGH than in the RES calves. A similar trend for higher adiponectin levels was reported in bull calves on a high preweaning level [49], as well as in ad libitum fed dairy heifer calves, accompanied by lower insulin concentrations in blood [34]. The greater concentrations of adiponectin in HIGH may point to higher insulin sensitivity in comparison to RES. However, we did not assess insulin sensitivity or responsiveness in the present study. Adiponectin was also suggested to have anti-inflammatory properties [50] and to exert a modulatory effect on oxidative stress [51]. However, when testing for correlations between the adiponectin and the Hp concentrations, there were no associations detectable. Considering the indicators for oxidative status tested herein, there were weak correlations between adiponectin and AOPP (µmol/g of protein; ρ = −0.135, *p* = 0.037) and dROM (ρ = 0.376, *p* < 0.001) detectable, but not between adiponectin, FRAP, and TBARS, respectively.

Circulating leptin parallels the body fat content and acts as a negative feedback regulator on feed intake [52]. However, for leptin neither the increase reported by Schwarzkopf et al. (2019) [16] between week 6 to week 20 of life for calves weaned in week 16 of life, nor a difference between the high and the restricted feeding level, as shown by Bruinjé et al. (2020) [53], was observed in our trial. The feeding group did not affect leptin values.

### 4.5. Oxidative Status

Oxidative stress is caused by an imbalance between pro and antioxidatives [54]. Due to the abrupt environmental change at birth, from hypoxic to hyperoxic, the cells of neonates generate reactive oxygen species (ROS) which can cause oxidative stress in neonates [17]. In the first sample collected from the calves herein (36–48 h p.n.), the relatively greatest values were observed for those variables that are indicative for oxidative damage of lipids (TBARS) and proteins (AOPP), whereas dROM increased with time reaching the greatest value around and after weaning. For FRAP, representing the antioxidant activity, no such patterns were clearly discernible. The activity of GSH‑Px, which couples the reduction of H_2_O_2_ with the oxidation of reduced glutathione as part of the enzymatic antioxidative defense, showed also the greatest values at first sampling (36–48 h p.n.) and a decline in the following weeks. Similar results were described by Ranade et al. (2014) [17], i.e., a decline of AOPP values from birth until week 18 of life, but the concentrations of dROM did not change in this study. Albera and Kankofer (2011) [18] reported that the total antioxidant capacity, based on FRAP measurements, at birth, 48 h later and on day 12 of life were on a similar level and higher than in their dams. The relatively higher values of TBARS and AOPP after birth might be related to the increased release of iron from fetal hemoglobin (Hb) [55] that is gradually replaced by adult Hb [56]. Iron is known for generating harmful oxygen species by promoting the Fenton reaction which produces the potent oxidant hydroxyl radical [55]. However, from this, also greater values of dROM at birth would be expected. AOPP values showing a decrease with increasing age supporting previous findings from Ranade et al. (2014) [17].

The underlying kinetics of the reactions related to the maturation of fetal Hb over time are unknown, and thus the results remain at the descriptive level. Group differences were limited to FRAP and AOPP. In case of FRAP, these differences were already apparent when the calves were not yet differing in feed intake (36–48 h p.n.). Potential differences in both the FRAP and the AOPP status of the dams were ruled out, and the respective values of the calves and their dams were also not correlated. Another reason might have been random differences in the redox balance of the colostrum provided, which may affect the oxidative status of the newborns and also their IgG uptake [57]. However, colostrum was not specifically analyzed in this regard. The reasons for this initial group difference thus remain unknown and the question, whether the continuation of the difference between groups is related to the feeding level or is a trajectory of the starting condition, cannot be answered. Concerning FRAP, the intake of antioxidatives, such as vitamin E, with MR was likely greater in the HIGH than in the RES group but it was the RES group that had elevated FRAP values. For AOPP, greater values in RES indicated higher portions of oxidized proteins, which are often functionally inactive and prone to hydrolysis but may also accumulate and cause further damages [58]. Regarding the activity of GSH-Px, the shift over time was comparable to AOPP and TBARS, but different to that of FRAP.

The weaning process can cause transient neutrophilia and suppress neutrophil phagocytic and oxidative burst responses in calves [40]. Weaning was mostly not associated with clear deflections in the curves of the indicators of oxidative status assessed herein. Only for FRAP, a distinct drop in concentrations was observed from week 14 to 16; thereafter the difference with greater values in RES tended to be reestablished. The AOPP values remained at the same level in both groups at that time. In a previous study of Ranade et al. (2014) [17], the dROM values in plasma did not change over the six-week-interval from birth until weaning at the age of 18 weeks, whereas in our study dROM increased with age.

## 5. Conclusions

High planes of MR feeding for 14 weeks improved the pre and the postweaning BW and ADG, without impairing the intake of solid starter feed. The reduced number of unrewarded visits at the feeding station in calves receiving 10 L/d compared to the restrictive feeding level (5.7 L/d) indicates that hunger as a sign of impaired welfare was alleviated by greater daily MR offers. Neither health status nor the Hp concentrations in serum were different between the feeding groups. The greater concentration of adiponectin, an insulin-sensitizing hormone, in HIGH-fed calves may reflect increasing insulin sensitivity, while leptin was not influenced by the feeding group. The patterns of various indicators of oxidative status were characterized throughout the first 20 weeks of life, but the results yielded no clear benefit or disadvantage for either group in terms of oxidative loads. Differences between feeding groups in FRAP were already apparent in newborns and thus might not be related to the MR feeding level. Greater AOPP concentrations in RES may be related to a loss of function of the oxidized proteins which in turn point to a need for degrading these proteins and synthesizing new ones, possibly resulting in greater protein turnover. It remains open to what extent the positive effects of HIGH feeding on BW and ADG would determine the future performance as a cow and influence the oxidative status.

## Figures and Tables

**Table 1:** Ingredients and composition of milk replacer (MR) and other feed.

Item (% of DM)	MR ^1^	Starter/Concentrate ^2^ (g/kg)	TMR ^3^
Grass silage	-	-	33.3%
Maize silage	-	-	20.7%
Hay	-	-	8.5%
Wheat straw	-	-	5.7%
Concentrate	-	-	31.8%
Chemical composition, % of DM			
DM, g/kg FM	-	-	49
CP	22	20	13.8
Crude fat	19	3.9	3.7
Crude fiber	0.1	5.2	21
aNDF_OM_^5^	-	-	43.8
ADF_OM_^5^	-	-	22.9
Ash	6.5	7.1	8.5
NFE ^6^	52.4	63.8	-
Total sugar	45.0	ND ^4^	-
ME ^7^, MJ/kg of DM	18.6	11.2	10.7
Ca	1	1	0.58
P	0.7	0.6	0.33
Na	0.4	0.4	0.1
Mg	ND ^4^	ND ^4^	0.18
K	ND ^4^	ND ^4^	18.5
Lysine	1.9	ND ^4^	ND ^4^
Methionine	0.7	ND ^4^	ND ^4^

^1^ MR ingredients: 50% skim milk powder, 24.5% whey powder, 17.5% vegetable oil, 2% glucose, 1.5% wheat soak powder, 0.5% whey protein powder (Milkivit Titan, Trouw Nutrition Deutschland GmbH, Burgheim, Germany). Additional MR ingredients per kg of DM: 200 mg Vitamin E; 150 mg Vitamin C; 75 mg Fe; 6 mg Cu; 85 mg Zn; 30 mg Mn; 1 mg calcium iodate; 0.3 mg Se (Milkivit Titan, Trouw Nutrition Deutschland GmbH, Burgheim, Germany). ^2^ Starter ingredients (per kg): 50 mg Vitamin A, Vitamin D3, and Vitamin E; 6 mg Cu, 40 mg Zn; 50 mg Fe; 40 mg Mn; 1.2 mg calcium iodate Ca; 0.2 mg Se; soya extraction meal, wheat bran, wheat, maize, wheat gluten, beet pulp, linseed, beet molasses, barley, calcium carbonate, sodium chloride, and monocalcium phosphate (Blattin Kälberstart Gold, Höveler Spezialfutterwerke GmbH & Co. KG, Dormagen, Germany). ^3^ Total mixed ration (TMR) fed after weaning from wk 14 of age onwards. ^4^ not determined (ND). ^5^ Neutral detergent fiber (aNDF) was assayed with a heat-stable amylase. aNDF and acid detergent fiber (ADF) are expressed as related to organic matter (_OM_), exclusive of residual ash. ^6^ Nitrogen-free extract (NFE), calculated as NFE = 100 − (CP + crude fat + crude fiber + ash); according to Frieten et al. (2017) [11] and the National Research Council [21]. ^7^ ME (metabolizable energy). The ME content of MR was calculated using the equation: ME, MJ/kg of DM = (24.2 × CP + 36.6 × fat + 17.0 × total sugar)/100 × 0.97 GE × 0.96 DE; according to Frieten et al. (2017) [11] and the National Research Council [21].

**Table 2:** Preweaning intakes (means ± SEM) of transition milk (whole milk of the dam, first five days of life) or/and milk replacer (MR), of starter, and of metabolizable energy (ME), total number of visits per day (no./d) at the automatic milk feeding station per day, as well as the number of unrewarded, rewarded visits, and drinking speed of calves with preweaning high allowance to MR (HIGH, 10 L/d) or restrictive allowance (RES, 5.7 L/d). The MR intake was gradually reduced in week (wk) 13 and 14 to 2 L/d in both groups. Pre and postweaning until wk 20 (means ± SEM) body weight and average daily gain (ADG) of these calves.

Variable ^1^	Feeding Group		SEM	*p*-Value		
	HIGH	RES		Group (G)	Week of Life (T)	G × T
Milk & MR intake, L/d	8.43	5.25	±0.09	<0.01	<0.01	<0.01
Starter intake, kg/d	0.77	0.86	±0.07	0.16	<0.01	0.75
Total ME intake, MJ/d	29.72	22.35	±0.64	<0.01	<0.01	<0.01
Total visits, no./d	12.07	20.91	±0.59	<0.01	<0.01	0.87
Unrewarded visits, no./d	5.28	15.72	±0.52	<0.01	<0.01	0.62
Rewarded visits, no./d	6.74	5.19	±0.14	<0.01	<0.01	0.27
Drinking speed, mL/min	488.68	479.00	±6.08	0.91	<0.01	0.68
Body weight, kg (wk 1–20)	107.86	99.14	±2.18	<0.01	<0.01	0.22
ADG, kg/d (wk 1–20)	0.98	0.89	±0.03	0.02	<0.01	<0.01

^1^ Values are presented as means ± SEM.

**Table 3:** Serum concentrations (means ± SEM) of haptoglobin (Hp), adiponectin, and leptin, and plasma concentrations (means ± SEM) of total protein content, advanced oxidation products of proteins (AOPP), thiobarbituric acid reactive substances (TBARS), reactive oxidative metabolites (dROM), ferric reducing ability of plasma (FRAP), and glutathione peroxidase activity (GSH-Px) in calves fed at a high level of MR (HIGH, 10 L/d) or a restricted level (RES, 5.7 L/d) and of their respective dams. The MR intake was gradually reduced in week (wk) 13 and 14 to 2 L/d in both groups. From week 15 until week 20 of life, the calves were moved to a new group pen in another stable receiving a TMR (Table 1). After birth/*post natum* is abbreviated as p.n. and after calving/*post partum* is abbreviated as p.p.

Variable ^1^	Feeding Group		SEM	*p*-Value		
	HIGH	RES		Group (G)	Week of Life (T)	G × T
**Haptoglobin (µg/mL)**						
Dams (36-48 h p.p.)	780.69	813.99	±175.39	0.58	-	-
Calves (36–48 h p.n.)	79.94	97.09	±14.91	0.47	-	-
Calves (wk 8–20)	37.92	38.89	±2.53	0.14	0.01	0.39
Calves (wk 1–20)	44.09	47.34	±3.54	0.10	<0.01	0.48
**Adiponectin (µg/mL)**						
Dams (36–48 h p.p.)	5.72	5.94	±0.58	0.81	-	-
Calves (36–48 h p.n.)	13.75	14.06	±0.63	0.73	-	-
Calves (wk 8-20)	15.41	14.38	±0.43	0.03	<0.01	0.67
Calves (wk 1–20)	15.18	14.33	±0.38	0.05	<0.01	0.55
**Leptin (ng/mL)**						
Dams (36–48 h p.p.)	21.84	22.93	±0.99	0.44	-	-
Calves (36–48 h p.n.)	4.48	4.09	±0.32	0.39	-	-
Calves (wk 8–20)	2.58	2.49	±0.05	0.17	0.03	0.74
Calves (wk 1–20)	2.83	2.72	±0.08	0.11	<0.01	0.82
**Total protein (g/L)**					-	-
Dams (36–48 h p.p.)	55.32	56.06	±1.89	0.79	-	-
Calves (36-48 h p.n.)	44.1	45.43	±1.11	0.40	-	-
Calves (wk 8–20)	49.39	49.81	±0.50	0.51	<0.01	0.85
Calves (wk 1–20)	48.68	49.19	±0.48	0.34	<0.01	0.89
**AOPP (µmol/g of Protein)**						
Dams (36–48 h p.p.)	0.82	0.88	±0.05	0.40	-	-
Calves (36–48 h p.n.)	1.95	2.40	±0.22	0.16	-	-
Calves (wk 8–20)	0.91	0.99	±0.03	0.03	<0.01	0.41
Calves (wk 1–20)	1.05	1.20	±0.06	0.01	<0.01	0.42
**TBARS (nM)**						
Dams (36–48 h p.p.)	-	-	-	-	-	-
Calves (36–48 h p.n.)	406.81	393.74	±21.47	0.67	-	-
Calves (wk 8–20)	243.75	246.19	±4.36	0.90	<0.01	0.19
Calves (wk 1–20)	266.83	266.43	±2.88	0.91	<0.01	0.23
**dROM (µg H_2_O_2_ equival./L)**						
Dams (36–48 h p.p.)	-	-	-	-	-	-
Calves (36–48 h p.n.)	17.11	19.02	±1.51	0.29	-	-
Calves (wk 8–20)	33.92	31.61	±1.11	0.11	<0.01	0.24
Calves (wk 1–20)	31.65	29.93	±1.09	0.28	<0.01	0.15
**FRAP (µM Fe^2+^/L)**						
Dams (36–48 h p.p.)	229.07	221.89	±8.55	0.55	-	-
Calves (36–48 h p.n.)	151.13	173.32	±7.43	0.05	-	-
Calves (wk 8–20)	161.77	168.32	±1.89	0.01	<0.01	0.12
Calves (wk 1–20)	160.35	169.05	±1.94	0.001	<0.01	0.10
**GSH-Px (U/mL)**						
Dams (36–48 h p.p.)	-	-	-	-	-	-
Calves (36–48 h p.n.)	0.011	0.012	±0.0006	0.42	-	-
Calves (wk 8–20)	0.01	0.01	±0.0002	0.69	<0.01	0.82
Calves (wk 1–20)	0.01	0.01	±0.0002	0.47	<0.01	0.86

^1^ Values are presented as means ± SEM.

## Data Availability

The data that support the findings and which are presented in this study are available on request from the corresponding author, Helga Sauerwein, sauerwein@uni-bonn.de. The data is not publicly available as not all data of the study has been published yet.

## Supplementary Materials

The following are available online at https://zenodo.org/record/4351318#.YA7rRhYxlPY, https://www.mdpi.com/2076-3921/10/2/260/s1, Table S1: Health parameters of weekly health checks.
