# Supplementary material for: Characteristics of the Oxidative Status in Dairy Calves Fed at Different Milk Replacer Levels and Weaned at 14 Weeks of Age
Source: Antioxidants (Basel). 2021 Feb 8;10(2):260. doi: 10.3390/antiox10020260 (PMC7915232; doi:10.3390/antiox10020260)
Supplement: Supplementary file 1 [file antioxidants-10-00260-s001.pdf]

Supplementary Table 1. Health parameters of weekly health checks.

Health score	1 = Completely healthy<br>2 = One health disturbance within one check and week<br>3 = Diseased and / or > 40°C rectal temperature (rectally)<br>4 > 39.5°C without any other symptoms / health disturbances
Health disturbances	1 = Rectal temperature > 39.5°C<br>2 = Digestion<br>3 = Respiration<br>4 = Eye-related<br>5 = Navel
Body posture	1 = lively, jumping around<br>2 = slightly subdued, standing<br>3 = subdued, chest position<br>4 = subdued, side position
Body temperature (rectal)	1 ≤ 38.0°C<br>2 = 38.1 – 39.5°C<br>3 = 39.6 – 40.0°C<br>4 ≥ 40.0°C
Navel inflammation	1 = Yes, 2 = No
Faecal consistency	1 = pasty<br>2 = thinly mushy<br>3 = soupy<br>4 = watery<br>5 = ruminant *faeces*
Abdominal rigidity	1 = soft<br>2 = tense, hardened
Apdominal spaltter / ringing	1 = negative on both sides<br>2 = positive on the left side<br>3 = positive on the right side
Depth of *bulbus oculi* in *orbita*	1 = 0 mm<br>2 = 1-2 mm<br>3 = 3-4 mm<br>4 ≥ 5 mm
Episcleral vessels filling	1 = moderately injected<br>2 = slightly injected<br>3 = moderate injected<br>4 = highly injected
Episcleral vessels sharpness	1 = sharp<br>2 = blurred
Heart rate	1 = 80 - 110/min<br>2 = 111 - 140/min<br>3 ≥ 140/min<br>4 ≤ 80/min
Colour mucosa (mouth)	1 = pink<br>2 = pale pink<br>3 = cyanotic<br>4 = white
Respiratory frequency	1 = 20 - 36/min<br>2 = 37 - 60/min

	3 ≥ 60/min
Dyspnoea (shortness of breath)	1 = none<br>2 = slight<br>3 = moderate<br>4 = high
Nares blown	1 = Yes, 2 = No
Mouth breathing	1 = Yes, 2 = No
Straight head-neck-posture	1 = Yes, 2 = No
